# Case Report: *de novo* in-frame deletion in *PLCG2* gene: a case report of B-cell lymphopenia, pulmonary bullae, and cutis laxa

**DOI:** 10.3389/fimmu.2025.1556372

**Published:** 2025-03-18

**Authors:** Xiaoqi Wu, Jingyuan Zhang, Min Shen

**Affiliations:** ^1^ Department of Rare Diseases, Peking Union Medical College Hospital (PUMCH), Chinese Academy of Medical Sciences & Peking Union Medical College; State Key Laboratory of Complex Severe and Rare Diseases, PUMCH, Beijing, China; ^2^ Department of Rheumatology and Clinical Immunology, Tianjin Children’s Hospital, Tianjin, China; ^3^ Department of Rheumatology and Clinical Immunology, PUMCH; National Clinical Research Center for Dermatologic and Immunologic Diseases (NCRC-DID), Ministry of Science & Technology; Key Laboratory of Rheumatology and Clinical Immunology, Ministry of Education, Beijing, China

**Keywords:** *PLCG2*, APLAID, B-cell lymphopenia, pulmonary bullae, cutis laxa

## Abstract

Phospholipase C gamma 2 (*PLCG2*) gene mutations might cause PLCG2-associated antibody deficiency and immune dysregulation (PLAID)/autoinflammation and PLCG2-associated antibody deficiency and immune dysregulation (APLAID) syndrome. They are two forms of autosomal-dominant immune dysregulation (ID). APLAID patients are usually characterized by skin lesions, pulmonary involvement, and musculoskeletal, ophthalmic, and gastrointestinal tract symptoms, but unlike PLAID patients, these patients do not present with cold urticaria or autoimmunity. Here, we report a 25-year-old man with B-cell lymphopenia, pulmonary bullae, recurrent sinopulmonary infections, and cutis laxa but without cold-induced urticaria. Anti-nuclear antibodies were negative. Trio whole-genome sequencing revealed a *de novo* heterozygous *PLCG2* gene (NM_002661.5) variant c.3417_3419del, p.E1139del, located on chromosome chr16-81973600-81973602. Our findings expand the variety of clinical and genetic phenotypes for APLAID and suggest that this variant would be meaningful.

## Introduction

Autoinflammation and *PLCG2*-associated antibody deficiency and immune dysregulation (APLAID) is a rare autoinflammatory disorder caused by *PLCG2* gene variants. PLCγ2 is a pleiotropic signaling molecule that controls cellular responses in many hematopoietic cells, including B lymphocytes and natural killer (NK) cells (1). PLCγ2 can hydrolyze the substrate phosphatidylinositol 4,5-bisphosphate (PIP2) to generate diacylglycerol (DAG) and inositol 1,4,5-trisphosphate (IP3). IP3 functions as a second messenger to increase the intracellular calcium concentration, inducing downstream cell activities (2–4). Pathogenic variants of the *PLCG2* gene can cause *PLCG2*-associated antibody deficiency and immune dysregulation (PLAID) or APLAID (5–7). Both are rare disorders inherited in an autosomal dominant pattern. Notably, their pathogenesis, along with clinical phenotypes and genotypes, are still being investigated.

Here, we report a rare case of APLAID that manifested primarily as pulmonary bullae and cutis laxa, with a *de novo* and novel in-frame deletion in the *PLCG2* gene.

## Case presentation

**Figure 1:**
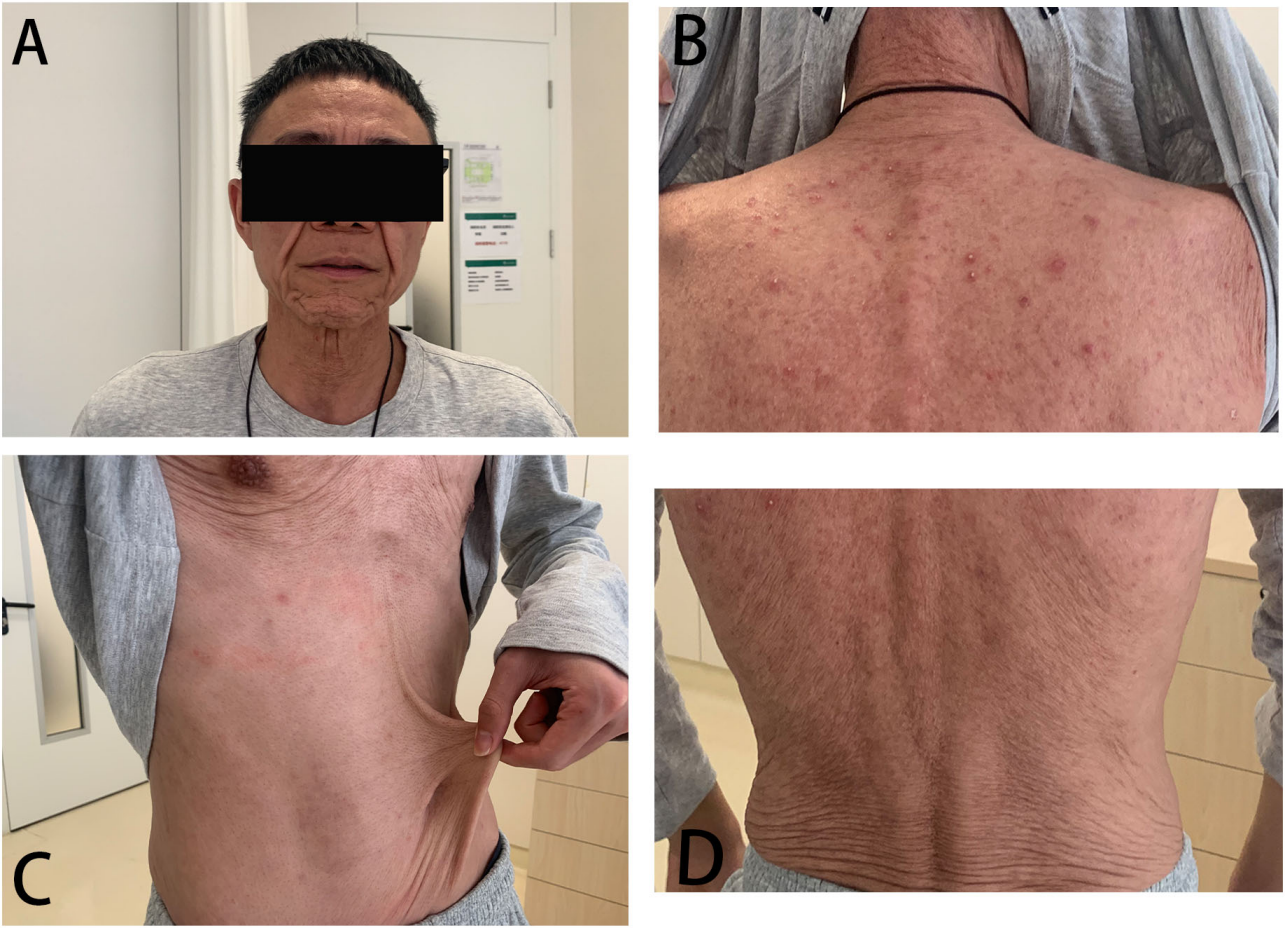
**(A-D)** Description of cutaneous manifestations in the patient. Cutis laxa on his face, neck, and abdomen. Red papules can be seen on his back, with pus-filled pustules in the center.

A 25-year-old Chinese Han man occasionally experienced chest distress and shortness of breath since the age of 3 years old. Pulmonary bullae were found through X-ray when he was 17 years old, and then, he experienced severe dyspnea exacerbations requiring hospitalization several times annually. He developed pulmonary *Aspergillus* infection when he was 20 years old and COVID-19 pneumonitis when he was 23 years old, leading to reduced exercise tolerance and frequent sinopulmonary infections requiring hospitalization and ICU (Intensive Care Unit) admission more than five times every year. At age 15, he underwent surgery for cryptorchidism and experienced fat liquefaction at the wound site, which persisted for more than a month. He also had cutis laxa ever since the age of 18, initially on the face and then subsequently spread to the trunk and extremities over 4–5 years. No cold-induced urticaria, abdominal pain, conjunctivitis, hearing loss, headache, prolonged fever unrelated to infection, or joint involvement was observed. The patient’s history of food and drug allergies was negative. He had no history of smoking or drinking. Family history was unremarkable. Physical examination revealed premature aging and obvious cutis laxa on the face, neck, and abdomen. Red papules can be seen on the back, with pus-filled pustules in the center (**Figure 1**). Lung examination revealed clear breath sounds bilaterally, with no dry or wet rales.

**Figure 2:**
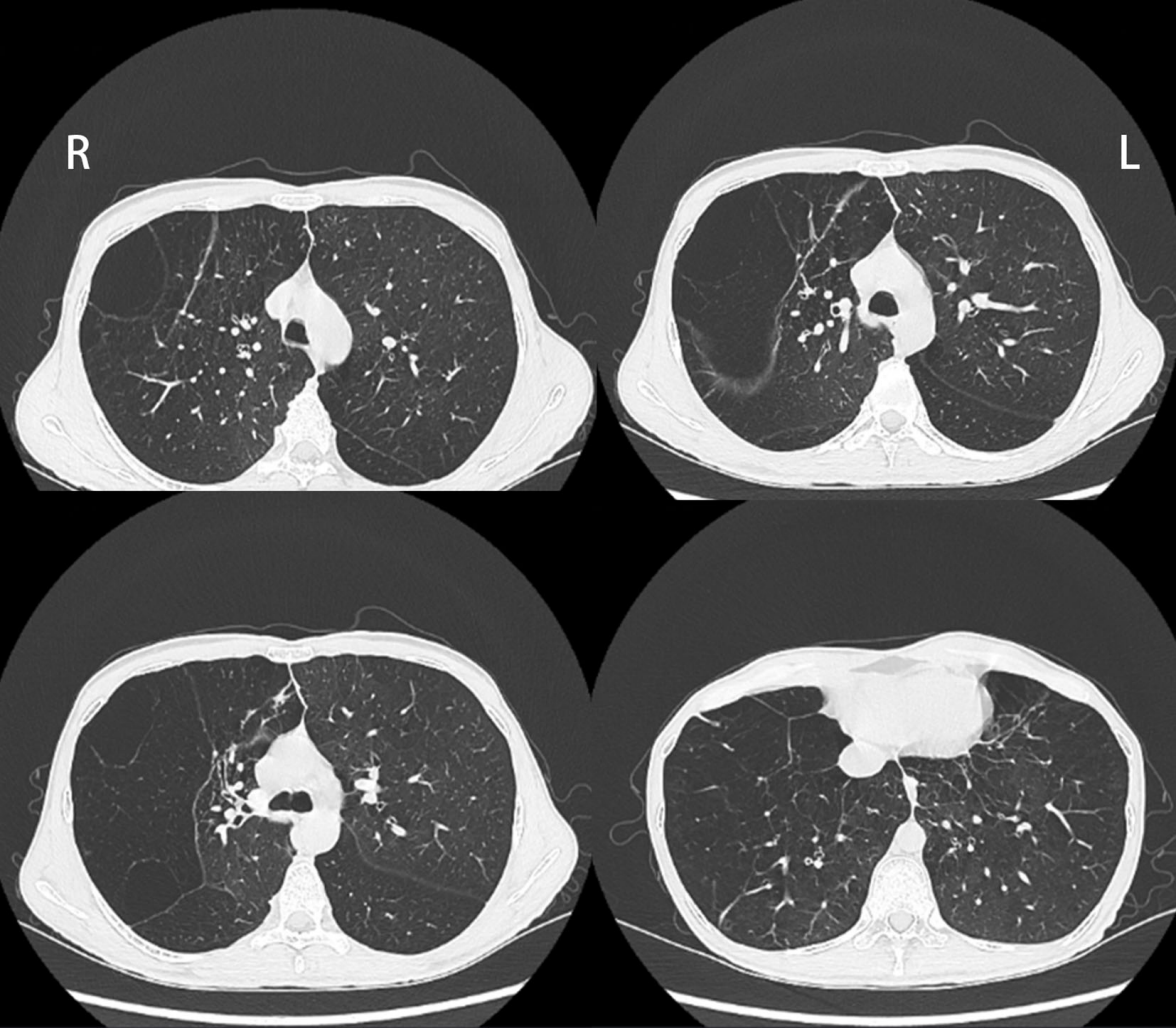
Chest CT showed bilateral emphysema and pulmonary bullae.

The laboratory results revealed normal T-cell counts, reduced circulating B cells 80/μL (normal range: 180–324/μL) and NK cells 160/μL (175–567/μL). The serum immunoglobulin (Ig) levels, including those of the IgG, IgA, IgM, IgE, and IgG subclasses, were within the normal limits. The neutrophil, eosinophil, and platelet counts and the serum levels of C-reactive protein (CRP) and complement were within the reference ranges. Serum protein electrophoresis and liver function tests were within normal parameters. Antinuclear antibodies (ANAs), lung cancer-related tumor markers and EBV-DNA were negative. Arterial blood gas analysis was normal. Skin biopsy revealed extensive infiltration of lymphocytes and neutrophils within the dermis, accompanied by loose dermal tissue and collagen fibers exhibiting curling and rupture upon elastic fiber staining, which was consistent with folliculitis and cutis laxa. A chest CT revealed bilateral emphysema and pulmonary bullae (**Figure 2**). The lung function measurements revealed extremely severe obstructive ventilation disorder, decreased diffusion capacity, and a negative bronchial dilation test. Trio whole-genome sequencing revealed a *de novo* heterozygous *PLCG2* gene variant (NM_002661.5): c.3417_3419del, p.E1139del, located at chr: 81973600 -81973602.

The presence of B-cell lymphopenia and recurrent infections, signaled underlying immune dysregulation, alongside skin lesions and a *PLCG2* gene mutation, leading to a diagnosis of APLAID in the patient. Budegolide aerosol or indacaterol combined with glycopyrronium had been previously utilized but was ineffective. He was given fluticasone inhalation powder once daily, 0.25 g azithromycin once daily for 3 days per week, regular bacterial lysates and vaccination (influenza/pneumonia vaccine). After the 6-month follow-up, the patient still had recurrent upper respiratory infections, but his overall condition was stable. The 6-minute walk test distance was approximately 400 m. The results of the reexamination of lung function were similar to those of the previous study. No obvious abnormalities were observed on the echocardiogram, and the systolic pulmonary artery pressure was 24 mmHg. The patient was suggested to continue with current treatment and to undergo lung transplantation if necessary.

## Discussion

We identified a novel in-frame deletion in *PLCG2* (c.3417_3419del, p.E1139del) within the C2 domain of an APLAID patient. While this specific variant (g.81973600_81973602delAGA) remains unreported, a proximal genomic deletion (g.81973597_81973599delAGA) resulting in the identical glutamic acid residue loss at position 1139 has been described (8). The reference sequence at chr16:81973594-81973606 is TGAAGAAGATATG. *In vitro* experiments have revealed that the E1139del mutation in PLCγ2 induces constitutive activation of the PLCγ2 signaling pathway in B cells, characterized by enhanced intracellular calcium flux and sustained ERK phosphorylation upon B-cell receptor (BCR) stimulation (8). It is a gain-of-function (GOF) mutation. The phred-scaled combined annotation-dependent depletion (CADD) of this variant is 22.8, and a score > 20 is in the top 1% of the predicted deleteriousness. Therefore, this variant has been predicted to be highly damaging. The patient with the *PLCG2* E1139 mutation, included in the previous study, presented with granulomas, recurrent sinopulmonary infections, hypogammaglobulinemia (low IgG levels), B-cell lymphopenia, and mildly reduced NK cell counts, but did not exhibit cold-induced urticaria. These findings showed similarities with our patient, suggesting the novel *PLCG2* c.3417_3419del mutation may contribute to this clinical phenotype.

Through a literature review, we found that most APLAID patients carried *PLCG2* missense variants, whereas in-frame deletions are always observed in PLAID (5, 6, 9). However, with continuous improvements in understanding the disease, some studies have described the p.Leu845-Leu848del variant in APLAID, whereas missense variants such as c.3249C>G (p.P1083=), c.3244T>C (p.C1082R), c.3524T>A (p.I1175K), and c.415C>T (p.P139S) are related to PLAID. In addition, the *PLCG2* variant c.77C>T, p.Thr26Met might cause phenotypical overlap of the PLAID and APLAID patterns (10–12). For the mutation region, the autoinhibitory C-terminal Src homology 2 (cSH2) domain is a critical site for the restriction of intrinsic enzyme activity, conferring GOF activity. Additionally, a key region linked to Ibrutinib resistance mutations in the PLCγ2 protein is located between amino acids 1139 and 1141 within the C2 domain, which is crucial for calcium binding and membrane attachment of PLCγ2. The *PLCG2* c.3417_3419del identified in our patient is also located in the C2 domain. Novice et al. (13) reported three patients with a novel heterozygous missense mutation in the C2 domain of PLCG2 (c. 3422 T>A, p.Met1141Lys), which have been shown to cause GOF effects, including hyperactive B-cell receptor (BCR) signaling and elevated calcium influx and two of the patients shared clinical features with APLAID. Thus the mutations in the C2 domain of *PLCG2* region may disrupt autoinhibitory mechanisms or calcium binding, leading to altered signaling. These data support an accurate diagnosis of APLAID in our patient, especially at the genetic level. As the E1139del mutation had been shown to cause GOF effects, so we did not conduct functional studies experiments in our patient.

Making a diagnosis of APLAID or PLAID is challenging because only 11 APLAID individuals with 7 variants of *PLCG2* have been reported thus far (5, 7, 9, 10, 13–15). It is not clear to what extent their clinical manifestations could differ from or overlap with those of other immune disorders. Similarly, the range of genetic changes has not been defined. APLAID patients always present with skin lesions, including cutaneous granulomas, vesiculopustular rashes and cutis laxa, posterior uveitis, inflammatory bowel disease (IBD) and recurrent sinopulmonary infections caused by a humoral defect, but with no circulating autoantibodies and have no cold-induced urticaria, in contrast to PLAID patients. On the basis of the above description combined with the patient’s clinical manifestations and genetic variation information, we diagnosed this patient with APLAID. The most common skin feature in APLAID is being vesiculopustular. However, our patient had rare cutis laxa, which has been previously reported in only three patients with APLAID (9, 10). Pulmonary bullae was the other major manifestation in our patient and have not yet been reported in APLAID. We ruled out infectious factors (such as tuberculosis) and lung cancer, as the patient’s serum protein electrophoresis was normal, the liver was not involved, and the genetic results did not indicate any mutations related to alpha-1 antitrypsin. Therefore, alpha-1 antitrypsin deficiency was also excluded from consideration. The patient experienced shortness of breath from childhood and pulmonary bullae were found before recurrent pulmonary infection, we think that this was probably related to autoinflammation in the lungs and might be one of the phenotypes of APLAID. But more case validation and further mechanistic research are needed to enhance our understanding of APLAID.

In APLAID, a disorder linked to pathogenic variants in the *PLCG2* gene, the phenomenon of B - cell lymphopenia is a key aspect of its immunological profile (5, 9).

PLC-γ2 is needed for efficient generation of memory B cells especially switched memory B- cell and that has no direct significant role in differentiation of germinal center B cells to plasma cells (16, 17).The relatively normal immunoglobulin (Ig) levels in APLAID can be attributed to the fact that the unaffected memory B cells and plasma cells can still contribute to immunoglobulin production. We regret that we did not perform a more detailed analysis of B lymphocyte subsets. Although the patient’s IgG levels were not low, he got recurrent sinopulmonary infections, so we recommended intravenous immunoglobulin (IVIG) therapy. However, he declined our recommendation due to personal reasons. Respiratory tract management, vaccination, and routine anti-infection treatment were provided to reduce infections while awaiting lung transplantation.

Overall, we suggest that the in-frame deletion mutation of the *PLCG2* variant c.3417_3419del p.E1139del might be causative for the APLAID phenotype in this patient. We hope that the present report adds more clinical and genetic evidence to the profile of APLAID.

## Data Availability

The raw data supporting the conclusions of this article will be made available by the authors, without undue reservation.
